# Phase Tandem Low-Coherence Interferometry for Surface Vibration Measurements

**DOI:** 10.3390/s25030681

**Published:** 2025-01-23

**Authors:** Petr Volkov, Alexander Bobrov, Alexander Goryunov, Mark Kovrigin, Andrey Lukyanov, Daniil Semikov, Oleg Vyazankin

**Affiliations:** 1 The Institute for Physics of Microstructures, Russian Academy of Sciences, 603087 Nizhny Novgorod, Russia; gor@ipmras.ru (A.G.); kovriginmark86@gmail.com (M.K.); luk@ipmras.ru (A.L.); semikovda@ipmras.ru (D.S.); vyazankin.os@ipmras.ru (O.V.); 2 Research and Educational Center, Physics of Solid State Nanostructures, Lobachevsky State University of Nizhniy Novgorod, 603950 Nizhniy Novgorod, Russia; bobrov@phys.unn.ru

**Keywords:** low-coherence interferometry, vibration measurements, phase interferometry, fiber-optic sensing

## Abstract

The development of optical methods for surface vibration measurements is currently of great interest. We propose a modified tandem low-coherence technique that utilizes the phase information of the low-coherence signal to detect surface vibrations. The resolution of this scheme is less than 1 nm for a 20 kHz bandwidth. The proposed technique is not just limited to measurements of surface vibrations, but it can also be used for interferometric fiber-optic sensors.

## 1. Introduction

Optical methods have become very popular for vibration measurements [1,2], with the main advantages of a lack of direct contact and a high sensitivity. There are a large number of different optical methods, for example, triangulation methods [3], which allow for the development of simple and sufficiently sensitive systems. Self-mixing interferometry [4,5] has become widespread but is sensitive to the feedback value and is also subject to additional noise, which limits its accuracy.

Interferometric techniques [6,7] have the highest accuracy, allowing for a subnanometer resolution and a wide frequency band, and, at the same time, they can be quite compact and relatively inexpensive. Interferometric methods use fluctuations in the optical path of the beam reflected from the measured surface. Two commonly used systems for interferometry are the Michelson interferometer, in which one of the mirrors is the surface under study, and the Fabry–Perot interferometer, in which one of the mirrors is the fiber tip, and the second is the surface under study. Modern processing methods have made it possible to restore the law of surface motion with accuracies of small fractions of a wavelength [8,9]. However, simple interferometric schemes have a major disadvantage—the sensitivity of the system depends on the position of the working point, especially in the case of small (in comparison to the optical wavelength) vibration amplitudes. There are a number of approaches to compensate for this effect. For example, in the spectral method, a spectrometer and a broadband source [10,11] or wavelength scanning laser [12,13] are used. Changes in the optical gap lead to a shift in the spectral fringes, thus allowing for vibration measurements; however, this method has a limited time performance.

There are various quadrature schemes [14,15], in which two sources are shifted along the wavelength or there is one source and a quadrature channel that is obtained by complicating the receiving circuit. In this case, the main advantage of interference schemes—the simplicity of implementation—is lost.

Various variants of homodyne techniques [16,17,18,19] are quite popular. In these variants, low modulation of either the source wavelength or the measured gap is used. A synthetic carrier is created, surface vibrations of which cause phase modulation of the interference signal.

A disadvantage of coherent measurements is that they measure with a discreteness of λ/2 due to the periodic nature of the interference signal. As a result, such schemes measure surface vibrations well but are not simultaneously able to obtain information about the absolute distance to the surface. Such information can be important, for example, when the measured object exhibits large displacements and additionally exhibits small fluctuations.

Low-coherence interferometry [20,21], which is based on the interference of light with a short coherence length, enables absolute measurements. Low-coherence interferometry (also called white-light interferometry) is mainly used in fiber gyroscopy and optical coherence tomography.

There are two main types of low-coherence systems that can be distinguished: systems with modulation of the optical path difference and systems with wavelength modulation. Although these two options are mathematically equivalent, they have significant differences in terms of technical implementation and the final parameters of the system. This paper will focus on a low-coherence system with modulation of the optical path difference. There are also a number of studies describing the use of low-coherence interferometry for detecting signals from fiber-optic sensors [22,23,24,25,26].

In this paper, a modification of tandem low-coherence interferometry is proposed, which allows us to simultaneously measure both the absolute distance to an object and small vibrations. Moreover, the proposed technique is not limited only to measurements of surface vibrations, but it can also be used for any interferometric fiber-optic sensors.

## 2. Materials and Methods

### 2.1. Phase Tandem Low-Coherence Interferometry

**Figure 1:**
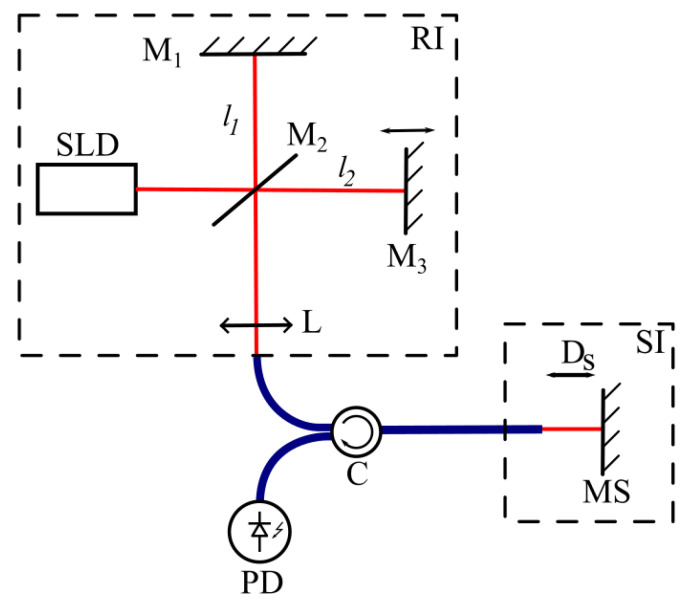
Scheme of TLCI. SLD—superluminescent diode; RI—reference interferometer; M_1_—fixed mirror of RI; M_2_—semitransparent mirror of RI; M_3_—scanning mirror of RI; L—optical lens; C—fiber-optic circulator; PD—photodiode; SI—sensor interferometer; MS—tested surface.

The proposed phase tandem low-coherence interferometry (PTLCI) method is based on tandem low-coherence interferometry (TLCI). The scheme of TLCI is shown in Figure 1.

A TLCI system consists of a pair of interferometers illuminated by a broadband source with a short coherence length. The first interferometer (reference) is able to adjust the arm length difference ($D_{r}=l_{2}-l_{1})$, while the second interferometer (sensor) has an arm length difference of $D_{s}$. Then, when the difference in the arm lengths of the reference interferometers changes, the intensity of the photodetectors will have the following form:

The maximum of the first pulse appears at the point $D_{s}=0$, and the maximum of the second pulse is at the point $D_{r}=D_{s}$. More information about TLCI can be found in [20]. If the power spectral density of the light source has the Gaussian form

(1) $$G\left(\nu \right)=\frac{1}{\sqrt{\pi {\left(\Delta \nu \right)}^{2}}}exp\left(-\frac{{\left(\nu -\nu_{0}\right)}^{2}}{{\left(\Delta \nu \right)}^{2}}\right),$$

where ν—the light frequency, $\nu_{0}$—the central frequency of the light spectrum, and Δν—the light spectrum width, which is well suited for superluminescent diodes. The TLCI signal will have the form

(2) $$I\left(D_{r}\right)=I_{0}\left(exp\left(-\frac{D_{r}^{2}}{L_{coh}^{2}}\right)cos\left(kD_{r}\right)+b\cdot exp\left(-\frac{{\left(D_{r}-D_{s}\right)}^{2}}{L_{coh}^{2}}\right)cos\left(k\left(D_{r}-D_{s}\right)\right)\right),$$

where $L_{coh}=c/\left(\pi \Delta \nu \right)$ is the coherence length of the light source, $k=2\pi /\lambda_{0}$ is the wave vector, $\lambda_{0}$ is the central wavelength of the light spectrum, $I_{0}$ is the light intensity, and *b* is a coefficient that depends on the reflection coefficients and insertion losses in the scheme.

**Figure 2:**
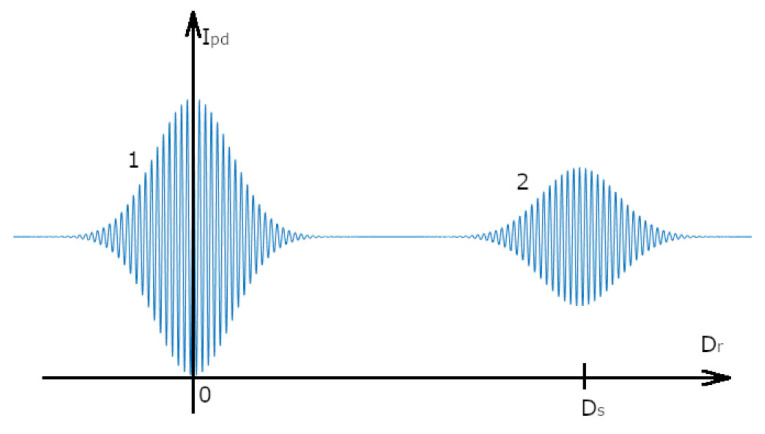
The intensity of the TLCI output. $D_{r}$—difference in the arm lengths of the reference interferometers; and $I_{pd}$—light intensity on the PD (Figure 1).

The first and second terms in (2) correspond to pulses 1 and 2 in Figure 2, respectively. Thus, if the value of $D_{r}$ is known at any time during scanning, then, since the maximum of the second pulse appears when $D_{r}=D_{s}$, this scheme enables remote measurements of an object’s thickness by determining the position of the maximum of pulse 2 in the scan.

This technique enables measurements of the absolute thickness of objects, but it is poorly suited for measuring objects whose thickness changes at high frequencies, since it requires a high-speed $D_{r}$ scanner. A modification of the TLCI method, which we call PTLCI and which enables measurements of fast processes, is proposed in this paper.

Let us look again at the scheme in Figure 1. Let the mirror in the reference interferometer (RI) move with a constant speed *V*; then,

(3) $$D_{r}\left(t\right)=D_{r0}+Vt,$$

where $D_{r0}$ is the starting point of scanning.

Let the thickness of the sensor interferometer (SI) vary as follows:

(4) $$D_{s}\left(t\right)=D_{s0}+∆D_{s}\left(t\right).$$

where $D_{s0}$ is a constant, $\left|∆D_{s}\left(t\right)\right|<L_{coh}$, and $\left|D_{s0}-D_{r}\left(t\right)\right|<L_{coh}$ Then, the light intensity at the photodiode, in accordance with (2), will be

(5) $$I=I_{01}exp\left(-\frac{{\left(D_{r}\left(t\right)-D_{s}\left(t\right)\right)}^{2}}{L_{coh}^{2}}\right)cos\left(k\left(D_{r}\left(t\right)-D_{s}\left(t\right)\right)\right).$$

where $I_{01}$ is a constant that depends on the reflection coefficients and insertion losses in the scheme. The first term in (2) will be negligible if $D_{s0},D_{r0}\;\gg L_{coh}$. Using (3) and (4), (5) can be modified to the following:

(6) $$I=I_{01}exp\left(-\frac{{\left(D_{r0}-D_{s0}+Vt-∆D_{s}\left(t\right)\right)}^{2}}{L_{coh}^{2}}\right)cos\left(k\left(D_{r0}-D_{s0}+Vt-∆D_{s}\left(t\right)\right)\right).$$

If the movement of the SI is slow in comparison to the scanning speed of the RI, i.e.,

(7) $$\frac{dD_{r}\left(t\right)}{dt}\ll V,$$

then (6) describes the narrowband signal. In this case, we can separate the slow envelope of the signal and the fast oscillation inside it. We can use any phase retrieval method to extract the phase of the signal (6), i.e., the expression under cos( ). Let us use the method based on the Hilbert transform [27,28]. If we have a narrowband signal of the form

(8) $$S\left(t\right)=A\left(t\right)cos\left(\omega t+\varphi \left(t\right)+\varphi_{0}\right),$$

where $A\left(t\right),\;\varphi \left(t\right)$ are slow varied functions in comparison with $cos\left(\omega t\right)$, then the Hilbert transform will modify such a signal to

(9) $$H\left\{S\left(t\right)\right\}=A\left(t\right)sin\left(\omega t+\varphi \left(t\right)+\varphi_{0}\right),$$

where $H\left\{\ldots \right\}$ denotes the Hilbert transform. Then, we can write that

(10) $$\Phi \left(t\right)=\omega t+\varphi \left(t\right)+\varphi_{0}=unwrap\left(arctan\left(\frac{H\left\{S\left(t\right)\right\}}{S\left(t\right)}\right)\right),$$

where *unwrap()* denotes the 2π step removing operation after the *arctan* operation. We have used the simple phase unwrapping algorithm [29]. When $\left|\Phi_{i+1}-\Phi_{i}\right|>\pi$, where $\Phi_{i}$ is the estimated phase at point *i* of the signal after discretization, we add or subtract 2π to or from Φ, depending on the difference sign. This method works well for signals with a good signal/noise ratio when the phase noise is small compared to π.

As with any phase reconstruction method, Hilbert transform-based methods have some error relating to the finite width of the signal spectrum and noise characteristics. Considerable information about this moment can be found in different works, for example, [28]. We will discuss the error of the proposed method in the next paragraph.

Thus, for signal (6), operation (10) will give

(11) $$\Phi \left(t\right)=k\left(D_{r0}-D_{s0}+Vt-∆D_{s}\left(t\right)\right).$$

The term $D_{r0}-D_{s0}$ is a constant phase shift and has no meaning in our example. The term *Vt* is the linear trend of the phase, determined by RI scanning, and the last term is the variation in the SI, which we want to measure. In the case of a highly stable RI scanning module, i.e., V = *const*, then the linear trend *Vt* can be simply removed. Thus, we will measure $∆D_{s}\left(t\right)$, which is of interest.

**Figure 3:**
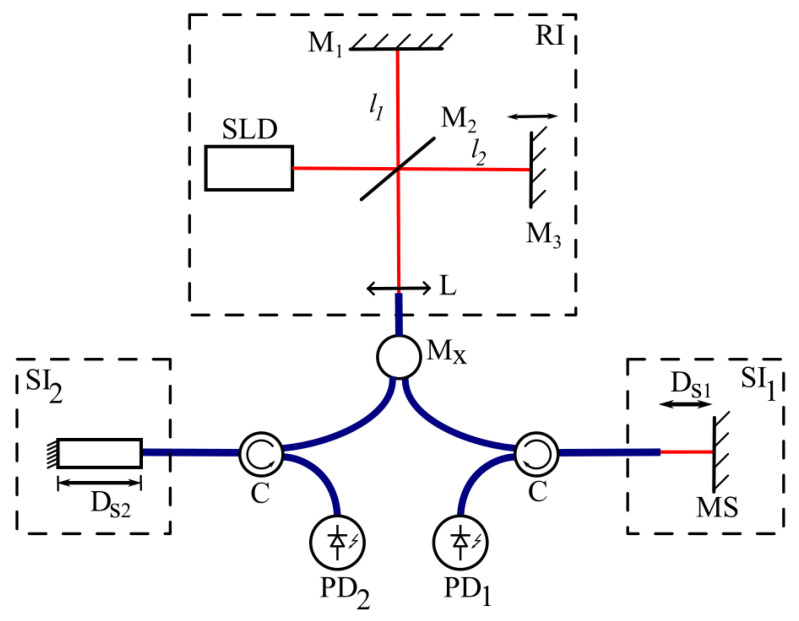
The modified scheme for vibration measurements.

The main problem for such a method is that, for real scanning devices, *V* may not be constant and may exhibit some variation, which will lead to phase errors in the measurements. In such cases, we can make some modifications to the scheme (Figure 3).

The scheme in Figure 3 has one RI and two SIs: one for vibration measurements and another to compensate for scanner instabilities. If

(12) $$V\left(t\right)=V_{0}+\Delta V\left(t\right),$$

where ΔV(t) is the scanner velocity variations, then let us make the second SI stable (for example, an intrafiber interferometer [25] with a stabilized temperature) with thickness $D_{s20}$. Then, the signals from photodiodes 1 and 2 will be, respectively,

(13) $$I_{1}=I_{01}exp\left(-\frac{{\left(D_{r0}-D_{s10}+V(t)t-∆D_{s}\left(t\right)\right)}^{2}}{L_{coh}^{2}}\right)\;\;\times cos\left(k\left(D_{r0}-D_{s10}+V(t)t-∆D_{s}\left(t\right)\right)\right),$$

(14) $$I_{2}=I_{02}exp\left(-\frac{{\left(D_{r0}-D_{s20}+V(t)t\right)}^{2}}{L_{coh}^{2}}\right)cos\left(k\left(D_{r0}-D_{s20}+V(t)t\right)\right),$$

where $D_{s10}$, $D_{s20}$—the constant part of thickness of the SI_1_ and SI_2_ in accordance with (4).

After phase extraction, we will have the following:

(15) $$\Phi_{1}\left(t\right)=k\left(D_{r0}-D_{s10}+V(t)t-∆D_{s}\left(t\right)\right),$$

(16) $$\Phi_{2}\left(t\right)=k\left(D_{r0}-D_{s20}+V(t)t\right).$$

Then, we can take the phase difference as follows:

(17) $${∆\Phi =\Phi }_{2}\left(t\right)-\Phi_{1}\left(t\right)=k\left(D_{s10}-D_{s20}+∆D_{s}\left(t\right)\right).$$

The term $D_{s10}-D_{s20}$ is a constant and can be adjusted to approximately zero by adjusting $D_{s20}$.

Thus, by the scheme in Figure 3, we can measure variations in the thickness of the sensor interferometer using the phase extraction algorithm. The proposed algorithm for PTLCI is mostly robust to light intensity variations because if these variations are slow in comparison with interferometric oscillations, then they will not affect the phase of the signal.

### 2.2. The Working Bandwidth of the PTLCI

To ensure the phase extraction method (10) works correctly, condition (7) should be met. Let us consider the harmonic oscillation of the SI:

(18) $$D_{s}\left(t\right)=D_{s0}+A_{s}cos\left(2\pi f_{s}t\right),$$

where $A_{s}$ is the amplitude of the oscillations, and $f_{s}$ is the frequency of the oscillations. Then, condition (7) will take the following form:

(19) $$2\pi f_{s}A_{s}\ll V.$$

We can also understand the limitations from the low end of the frequencies. The duration of the low-coherence pulse is about $2L_{coh}/V$; thus, the proposed method is well suited for frequencies that have more than one period during the pulse, i.e.,

(20) $$f_{s}>\frac{V}{{2L}_{coh}}.$$

**Figure 4:**
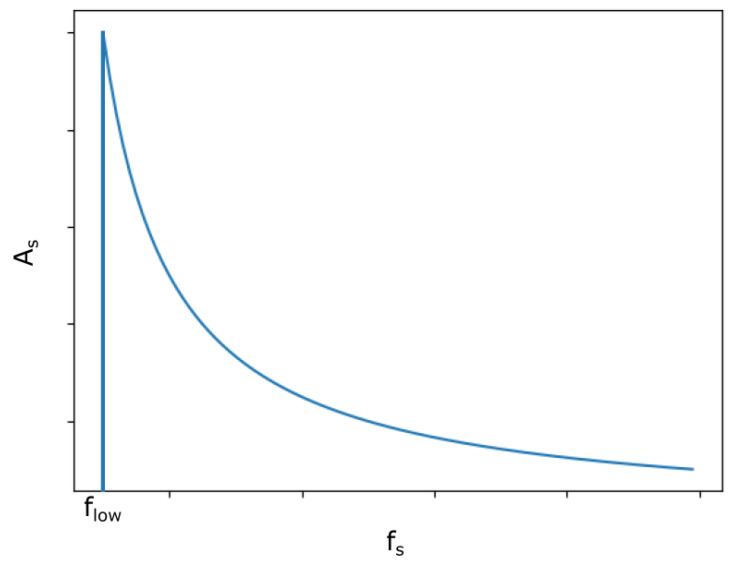
The working region of PTLCI. $A_{s}$—amplitude of the SI thickness oscillations; and $f_{s}$—frequency of the SI thickness oscillations in accordance with (18).

Thus, a qualitative view of the working region of PTLCI is shown in Figure 4, where the $f_{low}=V/\left(2L_{coh}\right)$:

For the typical parameters V=100 mm/s, $L_{coh}=25\;\mathrm{um}$, and $A_{s}=100\;\mathrm{nm}$, the frequency range will be $f_{s}>2\;\mathrm{kHz},\;f_{s}\ll 160\;\mathrm{kHz}$. The concrete upper limit for $f_{s}$ will depend on the required tolerance, as the tolerance of phase extraction depends on condition (7).

We can estimate a measurement error as the standard deviation of the detected signal from the original one over the entire sample length:

(21) $$err=\sqrt{\frac{\sum_{i}{\left(D_{s\_det}^{i}-D_{s\_real}^{i}\right)}^{2}}{n-1}},$$

where *i*—the index of the element in signal after discretization, $D_{s\_det}^{i}$—the detected SI thickness by PTLCI, and $D_{s\_real}^{i}$—real SI thickness.

**Figure 5:**
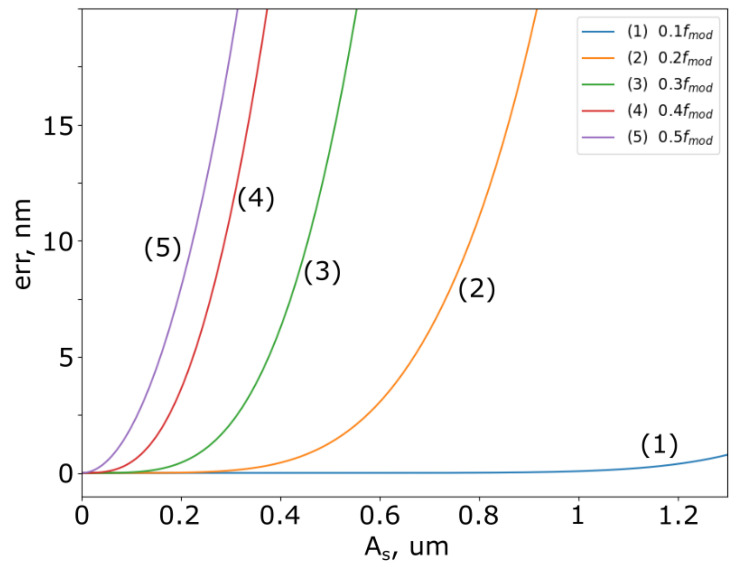
The calculated error for PTLCI.

Figure 5 shows the calculated dependence of the PTLCI error on the amplitude $A_{s}$ for five different frequencies $f_{s}$, $f_{mod}=V/\lambda$—frequency of interference signal, λ—the central wavelength of the light source. λ was taken as 1310 nm, which is typical for fiber optic schemes.

A very important feature of PTLCI is that the measured value in the method is the phase, which is uniquely related to the wavelength of the light as follows (considering that the light travels between the fiber tip and the mirror twice):

(22) $$∆d=\frac{∆\varphi }{4\pi }\lambda_{0}$$

where ∆d is the measured surface shift, ∆φ is the measured phase shift in accordance with (17), and $\lambda_{0}$ is the mean wavelength of the light source.

Thus, the proposed PTLCI method enables measuring the vibrations of sensor interferometers. It is important that SIs can be constructed by simply using the gap between the fiber tip and the surface (as shown in Figure 1), as this can be applied in any type of interferometric fiber-optic sensor.

## 3. Experiment

**Figure 6:**
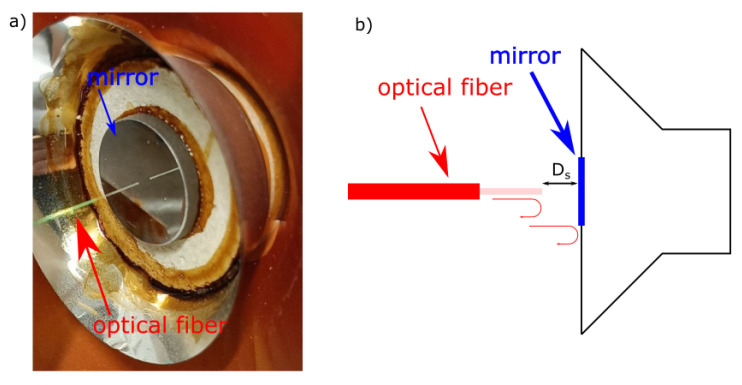
Sensing interferometer: (**a**) photo, and (**b**) scheme.

For the experimental proof of the proposed method, the scheme in Figure 3 was realized. The RI was constructed as a Michelson interferometer, where the scanning mirror was mounted on a magnetic movement system. SI_1_ was constructed using the gap between the fiber tip and the mirror and mounted on the acoustic speaker (Figure 6).

**Figure 7:**
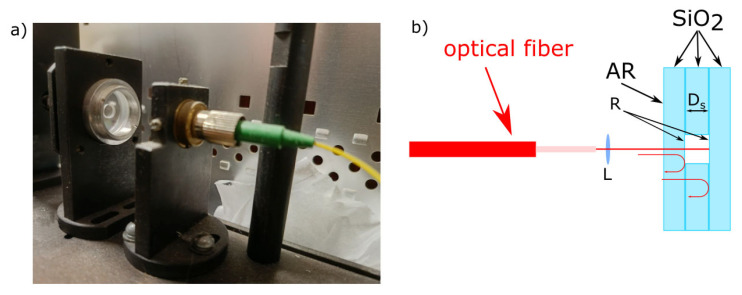
Sensing interferometer for modulator nonideality compensation: (**a**) photo, and (**b**) scheme. L—focusing lens; AR—antireflection coating; and R—semitransparent coating.

SI_2_, introduced for modulator nonideality compensation, was constructed according to Figure 7.

SI_2_ comprised a silica ring sandwiched between silica substrates with reflective coatings. Such a construction has low temperature drifts and a low sensitivity to external mechanical influences.

The light source was a superluminescent diode with a wavelength of 1310 nm, an optical power of 2 mW, and a spectral width of 40 nm. There are two waves in SI_1_ (Figure 6): the first one reflected from the fiber tip, and the second one reflected from the mirror. The reflection coefficient for the fiber tip is about 3.5%, and the reflection coefficient for the mirror is about 70%, but the optical power returned to the fiber decreased as the working distance increased due to the diverging beam of light emitted from the fiber. The optimal distance is the distance at which the intensities of the interfered waves are about the same. For SI_1_, this is about 1 mm. Note that the exact value of the working distance is not very important; it is important to satisfy the condition that the delay difference of SI_1_ and SI_2_ is small compared to the coherence length. Thus, both the distance from the fiber tip to the mirror in SI_1_ and the thickness of SI_2_ were about 1 mm. At the same time, increasing the distance from the fiber tip to the mirror in SI_1_ will lead to a decrease in the signal amplitude. Note that we can potentially use not only the mirror surfaces but also the diffusely reflecting surfaces. Of course, this will decrease the contrast of the interference and therefore the resolution; however, for the FOS, this usually results in a surface with good reflection.

**Figure 8:**
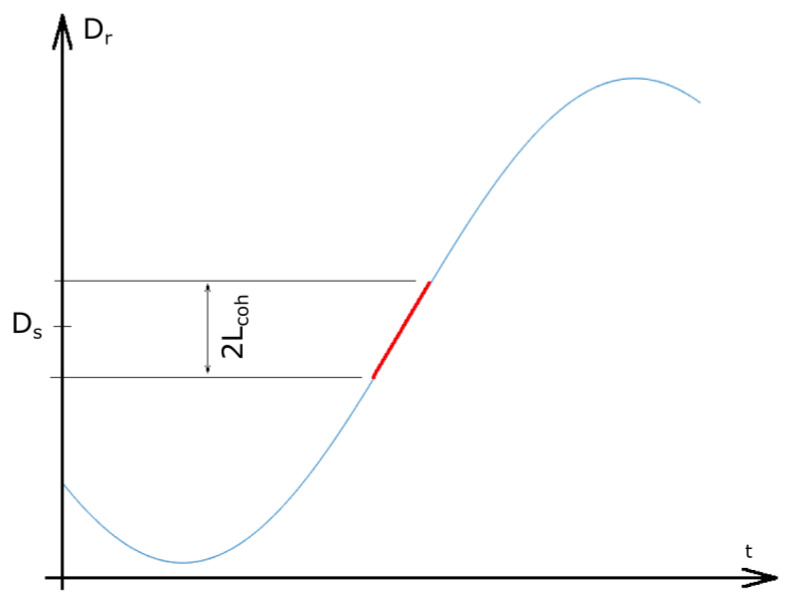
The working region of the RI. t—time; D_r_—difference in the arm lengths of the reference interferometer; and D_s_—difference in the arm lengths of the sensor interferometer. The red line highlights the working region.

The modulation signal of the scanning mirror in the RI was sinusoidal; however, the position of D_r_ = D_s_ was on an approximately linear part of the sinusoidal curve (Figure 8).

The modulation amplitude was chosen so that the frequency of the interference signal was about 100 kHz; thus, in accordance with (19) and (20), the working frequency range of the modulation was about 2–20 kHz.

**Figure 9:**
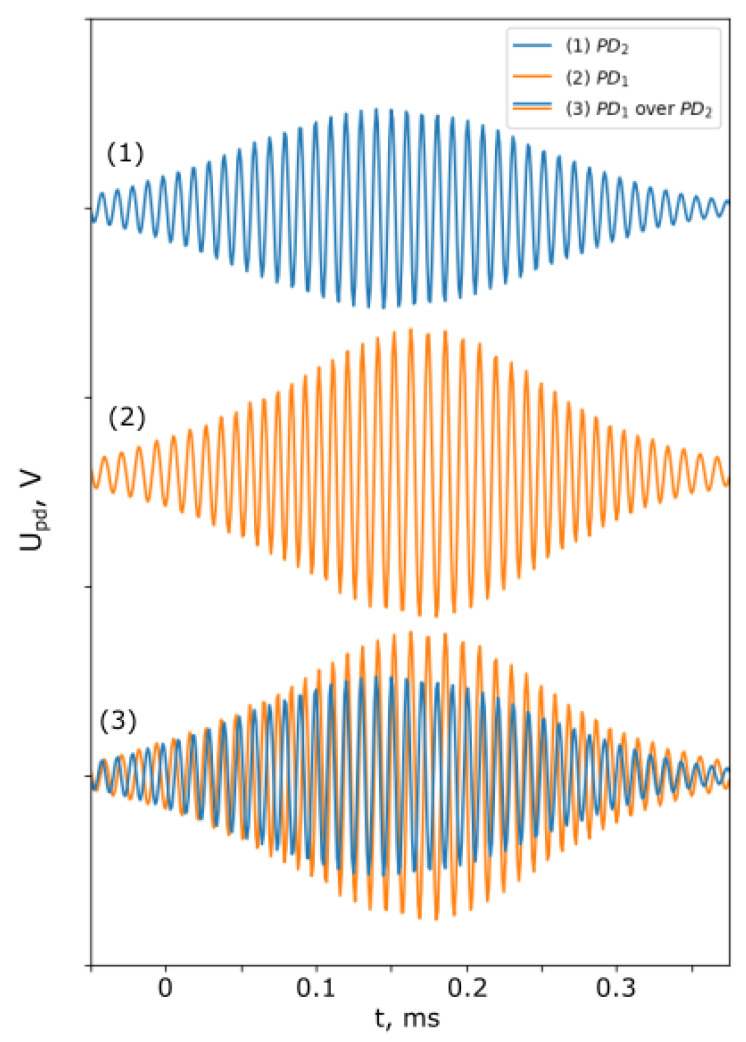
Signals from PD_1_ and PD_2_.

The mirror in SI_1_ was modulated with a sinusoidal signal with different amplitudes and frequencies, as in (18). Figure 9 shows the signals from PD_1_ and PD_2_ when $f_{s}=$ 5 kHz.

The moiré effect can be noted, which indicates the phase modulation of the signal at PD_1_.

**Figure 10:**
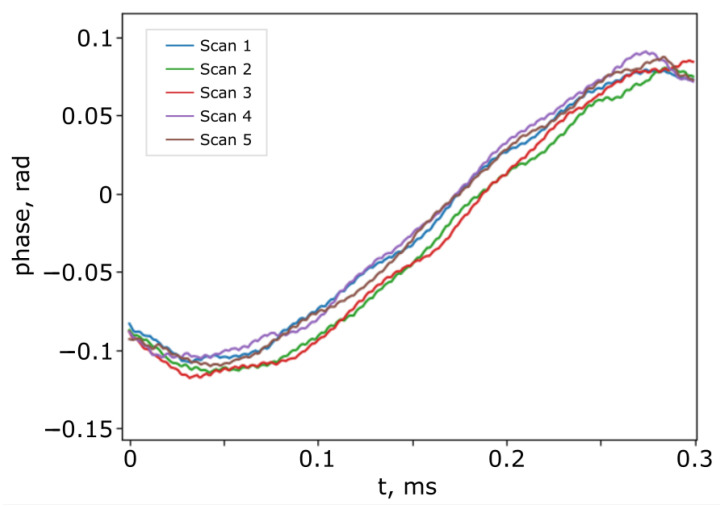
The difference in phase of the signals from PD_2_ for 5 sequential scans with the same driving voltage.

As mentioned above, the scanning velocity of the modulator may exhibit some instability. Figure 10 shows the phase extracted from the signal from PD_2_ for five sequential scans at the same driving voltage after the removal of the linear trend.

The instability of the scanner velocity leads to an additional phase shift of about 0.15 rad if we use the simple scheme in Figure 3 without SI_2_. The surface movement measurement error, in accordance with (21) and (22), is about 15 nm for $\lambda_{0}$ = 1310 nm. There is also some instability in the modulator from scan to scan.

The random phase shift from scan to scan is about 0.03 rad, which corresponds to an error of 3 nm according to (22).

**Figure 11:**
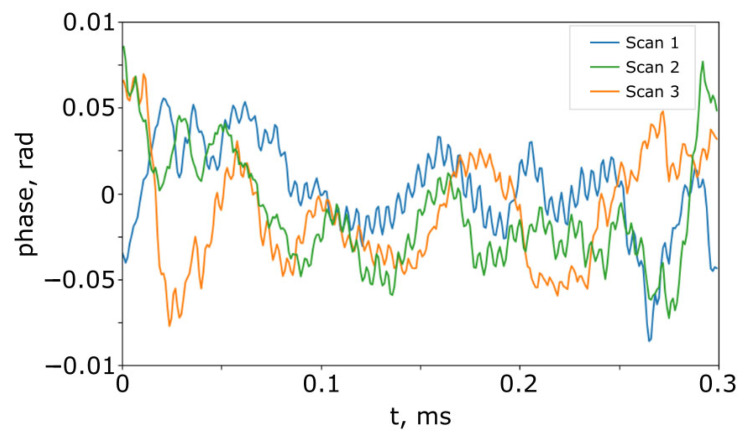
The difference in phase of the signals from PD_1_ and PD_2_ when SI_1_ and SI_2_ have the same construction as in Figure 7.

Using the stable SI_2_ to compensate for modulator nonideality eliminates this error. Figure 11 shows the phase difference in the three sequential scans when SI_1_ and SI_2_ are constructed, as shown in Figure 7.

It can be seen from Figure 11 that the modulator nonideality is compensated for and the system noise is less than 0.01 rad, which corresponds to an error of less than 1 nm according to (21). The oscillations of the extracted phase are typical for the Hilbert transform-based algorithms, but they are about an order less than measured phase fluctuations of the system.

## 4. Results

**Figure 12:**
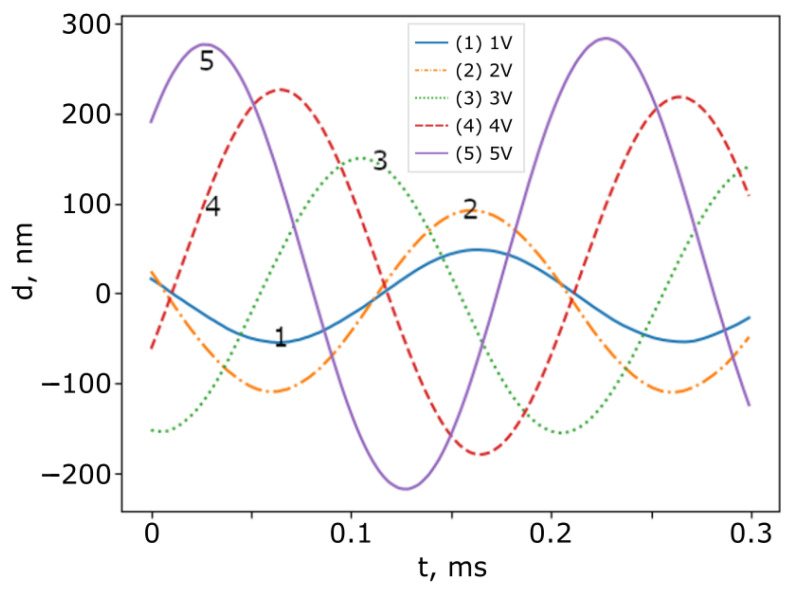
Measured surface vibrations with different driving amplitudes of the mirror in SI_1_. $f_{s}=$5 kHz. (1) $U_{m}$ = 1 V, (2) $U_{m}$ = 2 V, (3) $U_{m}$ = 3 V, (4) $U_{m}$ = 4 V, and (5) $U_{m}$ = 5 V.

**Figure 13:**
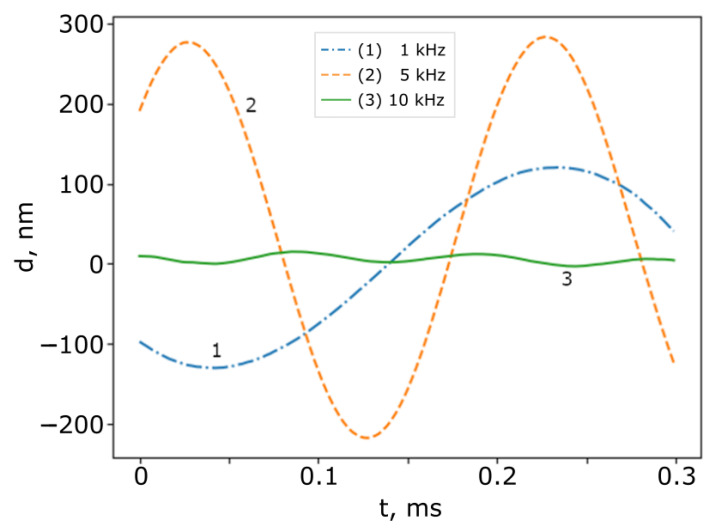
Measured surface vibrations with different frequency driving amplitudes of the mirror in SI1. $U_{m}$ = 5 V. (1) $f_{s}=1$ kHz; (1) $f_{s}=$ 5 kHz; and (2) $f_{s}=10$ kHz (3).

After testing the compensation algorithm, several measurements were performed. In the frequency range of 2 to 20 kHz, as mentioned above, the entire signal was filtered using a low-pass filter with a 20 kHz cutoff frequency. Figure 12 shows the measured surface vibrations of SI_1_ at different driving voltage amplitudes, $A_{s}$, of the mirror in SI_1_ when $f_{s}=$ 5 kHz, while Figure 13 shows the measured surface vibrations of SI_1_ at different driving voltage frequencies, $f_{s}$, of the mirror in SI_1_ when $A_{s}=$ 5 V.

It can be seen that the amplitude of the surface modulation increases monotonically as the driving voltage increases. This shows that, qualitatively, the proposed algorithm works correctly. We also can see that the amplitude changes with the frequency. This is due to the non-flat amplitude frequency response of the acoustic speaker, which was used to move the mirror in SI_1_.

**Figure 14:**
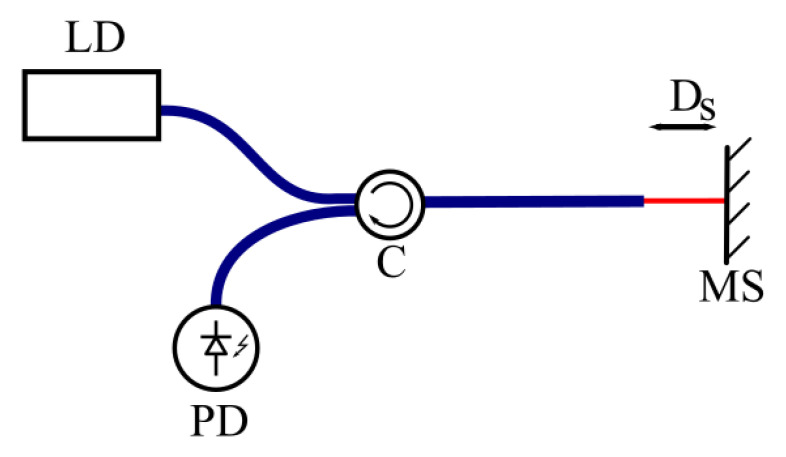
The scheme for dynamic calibration with laser interferometry. LD—laser diode; PD—photodiode; C—circulator; MS—vibrating surface.

To prove the tolerance of the method, we performed additional measurements of the mirror movement using classical laser interferometry using the simple scheme shown in Figure 14.

The calibration coefficient for the modulator can be defined as follows:

(23) $$\alpha =\frac{A_{s}}{U_{m}},$$

where $U_{m}$ is the driving amplitude of the modulator, and $A_{s}$ is the amplitude of the mirror displacement.

The signal from the PD for such a scheme will have the following form:

(24) $$U\left(t\right)=U_{0}+U_{s}cos\left(k(D_{s0}+A_{s}cos\left(2\pi f_{s}t\right))\right),$$

where $U\left(t\right)$ is the voltage signal from the PD and $U_{0},\;U_{s}$ are constants depending on the system parameters (optical reflection coefficients, optical insertion losses, PD response). It is clear that $U_{0}$ can be simply removed from the signal by subtracting the mean value of the signal.

**Figure 15:**
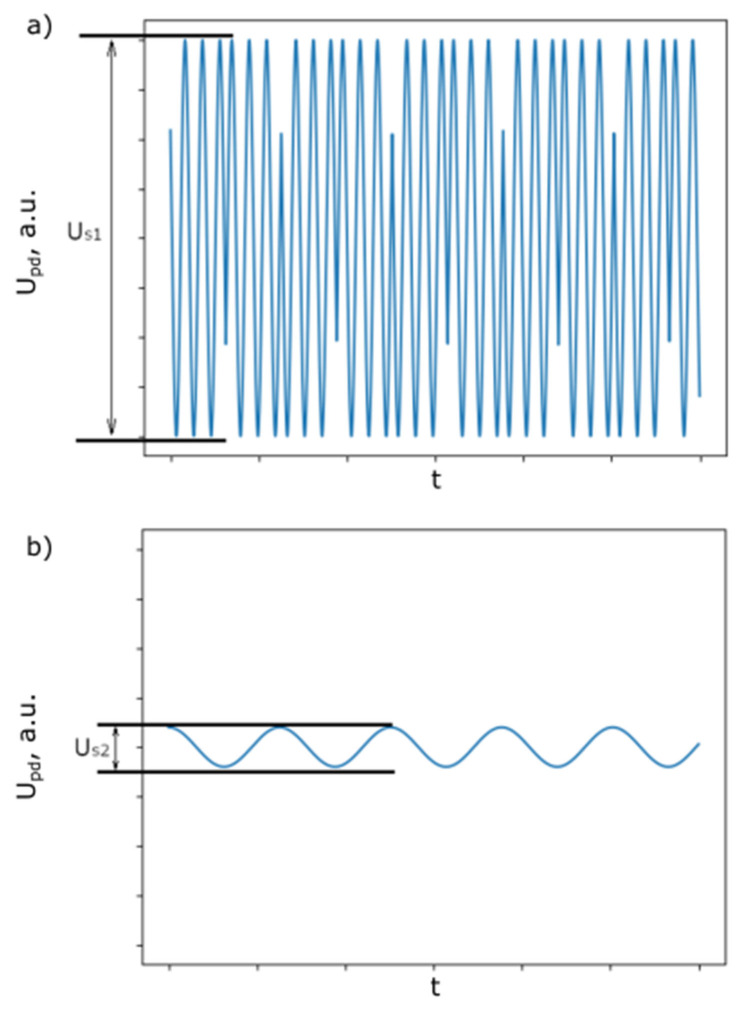
Calibration signals: (**a**) $A_{s}\gg \lambda /4$, and (**b**) $A_{s}\ll \lambda /4$.

Two measurements were then performed: the first with $A_{s}>\lambda /4$, and the second with $A_{s}\approx \;\lambda /40$ (Figure 15).

For the first measurement, in accordance with (24), the signal at the PD will have the form

(25) $$U\left(t\right)=U_{s1}cos\left(k(D_{s0}+A_{s}cos\left(2\pi f_{s}t\right))\right),$$

where the value $U_{s1}$ can be measured from the signal (Figure 15a). The term $U_{0}$ was removed.

For the second measurement, when $A_{s}\ll \lambda /4$ and $D_{s0}=(2n+1)\lambda /4$, which can be achieved by finely adjusting the modulator, the signal at the PD will be

(26) $$U\left(t\right)=U_{s1}sin\left(kA_{s}cos\left(2\pi f_{s}t\right)\right)\approx U_{s1}kA_{s}cos\left(2\pi f_{s}t\right)$$

Then, the amplitude $U_{s2}$, which also can be measured from the signal (Figure 15b), will be

(27) $$U_{s2}=U_{s1}\frac{2\pi }{\lambda }A_{s}$$

Then, using (23) and (27), the calibration coefficient for the modulator can be scored as follows:

(28) $$\alpha =\frac{\lambda \cdot U_{s2}}{2\pi \cdot U_{s1}U_{m2}},$$

where $U_{m2}$ is the amplitude of the driving voltage of the SI mirror at the second measurement. It can be seen from (28) that it is not important to know the exact value of $A_{s}$; it needs only to be small in comparison to λ/4.

**Figure 16:**
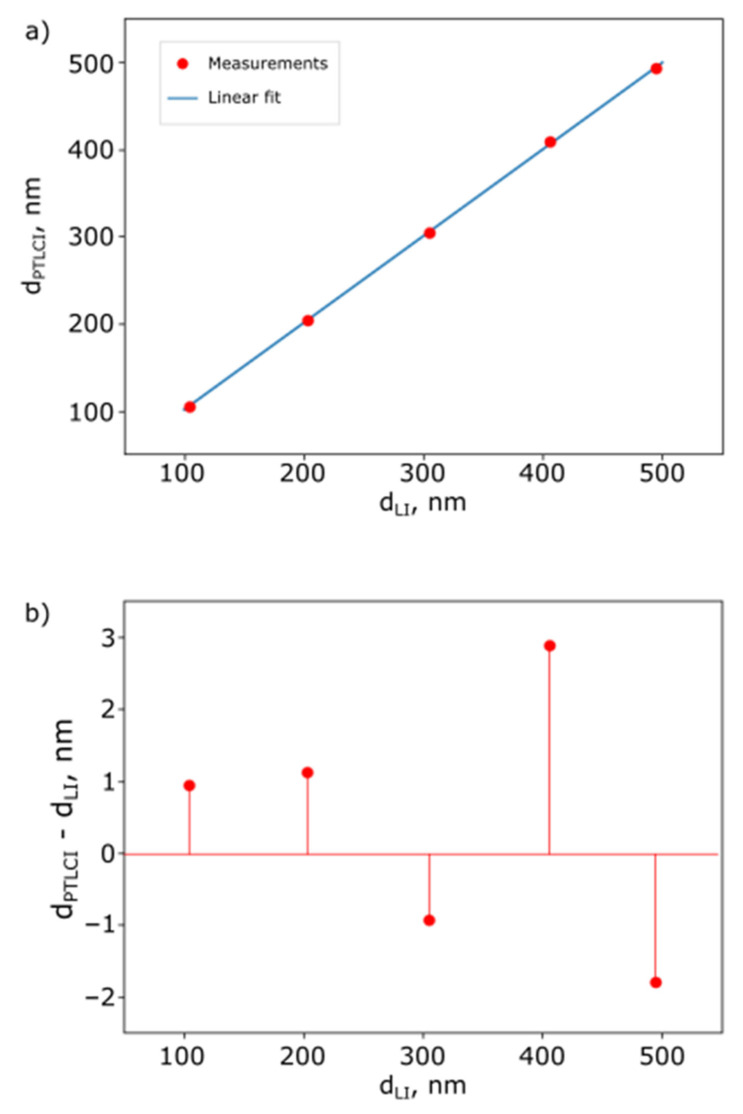
The relationship between PTLCI and calibration measurements: (**a**) measurements, and (**b**) difference.

Figure 16 shows a graph highlighting the relationship between PTLCI and calibration measurements for $f_{s}=$ 5 kHz.

It can be seen that there is very good agreement between PTLCI and laser interferometry. The difference in measurements is less than 3 nm. This error can be attributed as for the accuracy of the TLCI so for the acoustic modulator calibration. In any case, it can be determined that the measurement error of the mirror oscillation amplitude is less than 3 nm according to Figure 16 with the system’s resolution of less than 1 nm according to Figure 11. We also can see that the difference in the PTLCI results and the calibration increases with mirror modulation amplitude increasing. Apparently this is related to increasing errors of the used phase extraction algorithm based on the Hilbert transform. Nevertheless, the vibrations of the moving mirror are reliably detected up to small amplitudes.

## 5. Discussion

Optical methods for surface vibration measurements are being actively introduced into science and technology. Therefore, the development of these optical methods is very important. This article details a phase low-coherence interferometry method, which enables simultaneous measurements of the absolute distance to an object and control of small fluctuations.

This method is based on processing the phase modulation of low-coherence interferometric signals, which occurs when the measured surface fluctuates. This leads to the phenomenon of the measurement result not depending on the reflection coefficients of the surface nor on the random insertion losses in the optical circuit. At the same time, it is possible to achieve a resolution of less than 1 nm in the 20 kHz frequency band.

The main limitations of the proposed scheme are two-fold: firstly, measurements cannot be carried out continuously, and, secondly, the operating frequency range is limited by conditions (19) and (20) and spans one order of magnitude.

The first disadvantage can be eliminated by introducing two scanning interferometers operating with a modulating voltage phase shift in π/2. Given that the modulation range is quite low, such modulators are easy to implement using optical fibers and are also compact and cheap.

The second disadvantage, on the one hand, limits the parameters of the system, but, on the other hand, it leads to a number of possibilities. By changing the scanning speed in the reference interferometer, the frequency operating range of the system can be shifted at any time, allowing for an adaptive system. Moreover, since a method to compensate for the imperfections of the reference interferometer’s modulator was implemented, the modulation system is as simple as possible while still operating in a wide frequency range. This can be most easily implemented using fiber-optic circuits.

The proposed variant is interesting when you need to measure the vibration of the surface, which has, at the same time, the directional movement. It allows us to have a low-speed optical path difference modulator to measure high-frequency vibrations.

Another important point is that the described technique is not limited to measuring surface vibrations but can also be applied to any interference fiber-optic sensors.

## Data Availability

All evaluated data are presented in this paper in graphical form. The raw measured data of this study are available upon request from the corresponding author.
